# Effect of Harvest Date on Fruit Quality and Post-Harvest Storability of Three Different Peach Cultivars

**DOI:** 10.3390/foods15030421

**Published:** 2026-01-23

**Authors:** Belén Velardo-Micharet, Marisol Duarte-Maya, Ana Cristina Agulheiro-Santos, María Concepción Ayuso-Yuste, María Josefa Bernalte-García

**Affiliations:** 1 Technological Institute of Food and Agriculture of Extremadura (CICYTEX), Avda. Adolfo Suárez s/n, 06007 Badajoz, Spain; 2 MED-Mediterranean Institute for Agriculture, Environment and Development & CHANGE-Global Changeand Sustainability Institute, IIFA-Institute for Advanced Studies and Research, University of Évora, Pólo da Mitra, Ap. 94, 7006-554 Évora, Portugal; acsantos@uevora.pt; 3 Department of Plant Science, School of Sciences and Technology, University of Évora, Pólo da Mitra, Ap. 94, 7006-554 Évora, Portugal; 4 Agriculture Engineering School, University of Extremadura, Avda. Adolfo Suárez s/n, 06007 Badajoz, Spain; 5 University Research Institute for Agricultural Resources (INURA), University of Extremadura, 06007 Badajoz, Spain

**Keywords:** *Prunus persica*, modified atmosphere, ripening, cold storage, shelf-life

## Abstract

Peach consumers demand good quality fruit, but premature harvests result in fruit that does not ripen properly and does not reach the required organoleptic quality, so consumers stop buying this product that does not meet their expectations. In our region, peaches are exported long distances, and it is required that when they reach the destination market their quality is adequate. Therefore, the objective of this study was to determine the storage capacity of commercial and delayed harvest in three peach cultivars. ‘Rich Lady’, ‘Summer Lady’, and ‘Merryl O’Henry’ were harvested at commercial maturity (H1) and, a few days later (H2), packed in passive modified atmosphere (PMA), and stored under refrigeration for up to 40 days to simulate marketing to distant markets. During storage and after three days of shelf-life, the physico-chemical characteristics, damage, and sensory quality of the fruit were analyzed. In general, after cold storage, peaches improve their sensory characteristics after three days at room temperature. PMA with refrigeration was suitable for exporting ‘Rich Lady’ peaches overseas for H1. The late harvest, H2, is recommended for ‘Summer Lady’, as it improves sensory quality without losing storability. ‘Summer Lady’ was the best-rated cultivar by the tasters, and ‘Merryl O’Henry’ the worst, due to its lack of ripening and high incidence of chilling injury.

## 1. Introduction

Peach [*Prunus persica* (L.) Batsch] is native to western and central China, where the species still shows the greatest diversity [1], and currently this country is the main peach producer, with more than 64% of the world total. It is a very important crop in the European Union, with around 27 million tons being produced annually, with Spain being the second largest producer of peaches and nectarines, with 1382 thousand tons produced in 2023 [2]. In Spain, it is grown in different regions, which allows for a wide harvest calendar, and it is a strategic sector that has undergone an important varietal renewal of plantations. In recent years, much of the stone fruit production in Spain is destined for export, so it is of great economic interest [3]. Therefore, it is necessary to know the ability of cultivars for marketing over long distances, maintaining their quality.

Peach is a climacteric, soft-fleshed, highly perishable fruit, so it is essential to determine the harvest date, to collect the fruit at the optimum point of maturity, and thus to achieve its proper conservation and long-distance shipping. A previous study conducted with Japanese plums determined that the harvest date has a significant influence on quality parameters and post-harvest damage, and therefore on long-distance marketability [4]. This will ensure that their organoleptic characteristics are optimal for consumption when fruits arrive at their destination [5]. In early harvests, peaches cannot complete the climacteric evolution during storage, and the fruits will have poor organoleptic quality, which leads to frequent consumer complaints [6]; immature fruits are also more susceptible to dehydration and internal damage [7]. On the other hand, in late harvests, fruits are less firm and more susceptible to decay [4,8].

In commercial practice, peaches are pre-cooled and kept refrigerated at a constant temperature when they arrive at the fruit facility to extend their commercial life. Variations in storage temperature, or temperatures within the range of 2.2 to 7.6 °C, favor the development of physiological disorders, known as chilling injuries (CIs) [9]. Susceptibility to CI depends on the cultivar, but there are other factors such as storage time, fruit size, tree load, or season that are not yet well understood [10,11]. CI often manifests itself after refrigeration, during shelf-life, and usually results in texture problems such as mealiness, leatheriness, and internal browning, among others, which reduce commercial quality, and negatively affect consumer perception [11,12,13].

Passive modified atmosphere (PMA) is used as a complementary technique of refrigeration for peach exports to distant markets [7,14,15], which allows for maintaining quality for a longer time, slowing respiration rate, and has an inhibitory effect on post-harvest pathogens [16,17,18]. Lara et al. [19] observed in peaches subjected to a low-oxygen atmosphere that ripening is delayed, and that changes in different metabolic pathways are important but generally reversible.

During marketing at room temperature, the fruit completes its organoleptic ripening, but this is when chilling injury and other damages are revealed [11,13,20]. Pre-storage at room temperature, from 24 to 48 h, prior to the application of PMA, has been shown to be effective in reducing chilling injury in ‘Douradão’ peaches, maintaining other quality traits during the simulated marketing period of the fruit [20].

Consumers appreciate peaches for their flavor and aroma, sweetness, firmness, juiciness, color, and damage absence, which are now considered relevant issues in breeding programs [8,12,21]. Initial purchases are related to appearance, while repeated purchases are based on good eating quality; so sensory properties play a relevant role in consumer preference and purchasing loyalty [22,23,24]. Long-term storage and post-harvest studies must take into account the evaluation of consumers, for whom the product is destined [25]. The most frequent consumer complaints are hard fruit and a lack of flavor at consumption, caused by too early harvesting [8]. Peach quality is not always homogeneous, as there is a wide diversity of cultivars with different organoleptic characteristics, and sometimes, attractive fruits are of low eating quality, so these problems can lead to decreased consumer acceptance and a reduction in sales and consumption [24,26].

The aim of this study was to determine the effect of two harvest dates, commercial and slightly delayed, on the marketability to distant destinations of three peach cultivars of high export interest. For this purpose, long-distance commercialization was simulated using refrigeration and PMA. The effect on physico-chemical parameters, damage incidence, and sensory quality were studied throughout refrigerated storage and subsequent shelf-life.

## 2. Materials and Methods

### 2.1. Plant Material

Three peach [*Prunus persica* (L.) Batsch] cultivars of commercial interest (‘Rich Lady’, ‘Summer Lady’, and ‘Merril O’Henry’) were employed. Each was supplied by a different fruit center located in the province of Badajoz (South West of Spain). The three orchards were very close to each other, so the soil and climate conditions were very similar, as well as the farm management practices used. The peaches have a red epidermis and yellow flesh, and are usually harvested at the end of June, end of July, and mid-September, respectively (Supplementary Figure S1).

Field technicians at the fruit centers defined the date of harvest at commercial maturity for overseas export (H1), with a penetrometer firmness between 3.5 and 4.5 kg, and TSSs > 10–12 °Brix. There was a second harvest (H2), with fruit firm enough for handling, which took place some days after H1: 5 days later for ‘Rich Lady’, 6 days for ‘Summer Lady’, and 8 days for ‘Merryl O’Henry’.

### 2.2. Experimental Design

Once harvested, the peaches were immediately taken to the fruit plant and handled according to standard procedures; the fruits were placed in boxes (3 for each sample date) with 60 pieces each, distributed in two heights by means of alveolus sheets and packed in a passive modified atmosphere (PMA) using X-Tend^®^ plastic bags (StePac L.A Ltd., Tefen, Israel). Then, they were transported to the laboratory (CICYTEX-INTAEX, Badajoz, Spain) in a refrigerated vehicle, so as not to break the cold chain. Post-harvest storage was carried out by simulating overseas transport conditions (0 °C, without relative humidity control), assuming a maximum transport period of 40 days at the furthest possible destination. After refrigerated storage, X-Tend bags were opened and the boxes were transferred to a chamber at 20 °C for three days (shelf-life). Fruit quality parameters were analyzed at harvest (0), after 10, 20, 30, and 40 days of cold storage and after shelf-life (10 + 3, 20 + 3, 30 + 3, and 40 + 3 days).

### 2.3. Methodology

#### 2.3.1. Pomological Characterization

Weight and size were determined at H1 and H2, with the fruit arriving directly from the field, without previous preparation, using 30 fruits from each harvest (*n* = 30). The weight of each peach was determined on a Metter Toledo PB 1502 electronic balance (Metter Toledo AG, Greifensee, Switzerland) with an accuracy of 0.01 g, and for size determination, the axial and equatorial diameter of the same fruit was measured with a 0–150 mm digital caliper (Infoagro Systems S.L, Madrid, Spain).

#### 2.3.2. Physiological and Physico-Chemical Determinations

Weight loss was calculated as the difference between the initial and final weight of the fruit (*n* = 30), after refrigerated storage and shelf-life, and the results were expressed as a percentage.

The concentration of O_2_ and CO_2_ inside the package was measured with a portable gas meter Oxibaby 6 (Witt Gasetechnic, Santander, Spain) [27]. These measurements were carried out daily until the equilibrium modified atmosphere was reached and, from that moment on, the composition of the atmosphere was measured during storage and at each date of analysis, i.e., at 10, 20, 30, and 40 days of refrigerated storage, in three different boxes (n = 3), and results were expressed as a percentage.

Firmness was determined by penetration test using a TA-XT2i Texture Analyzer (Aname, Pozuelo, Madrid, Spain). For each sample, peaches were tested twice at distally opposite sites, in the equatorial zone, without epidermis. For this purpose, a cylindrical probe of 8 mm diameter was introduced to a depth of 8 mm, on 20 fruits for each treatment and date of analysis. Firmness was expressed as the maximum force (N) of the force/deformation curve.

The instrumental measurement of epidermis color was carried out with a Minolta Chroma Meter CR-400 (Aquatecnika, Valencia, Spain), by reflectance on an area of 8 mm in diameter with illuminant D65 and a vision angle of 0°. For each treatment and date of analysis, 20 peaches were measured at two diametrically opposite points on the equator of each fruit, one measurement on the sunny side and the other on the shaded side. The coordinates L* (brightness, white–black), a* (green–red), and b* (blue–yellow) were obtained and then were calculated as follows: Chroma (C*), Hue angle (H) and, CIRG index (Color Index Red Grape, CIRG= (180 − H*)/(L* + C*)) [28].

Total soluble solids (TSSs) were measured by a digital refractometer Pal-1 (Atago CO., LTD, Tokyo, Japan) and expressed as °Brix (*n* = 20). For this purpose, the 20 fruits that had already been employed for firmness were used by cutting the fruit in half and squeezing out the juice. The remaining half was used for sensory evaluation, so that the three measurements were performed on the same fruits.

To determine the acidity (TA), 5 homogenates (*n* = 5) were prepared with three peaches each, using a domestic blender and then an Omni Mixer (Waterbury, CT, USA). It was determined with an automatic titrator Mettler Toledo T-50 Graphix (Metter Toledo, Coslada, Madrid, Spain), using NaOH 0.1 N solution, and the results were expressed in g of malic acid per 100 g of fresh sample.

Acceptability index ratio (AI) was calculated as the ratio between total soluble solids and acidity (TSSs/TA).

#### 2.3.3. Post-Harvest Damage Assessment

Damage assessment was carried out visually, first in the epidermis and then in the flesh of the peaches (*n* = 20). The following types of damage were evaluated: mechanical damage, chilling injuries, dehydration, decay, and split-pit incidence. The total number of damaged fruits was counted and the results were expressed as a percentage [4], and the intensity of damages was determined for each fruit according to the following scale [29]: 1 Healthy: No damage, 2 Slight: 0–25% of the fruit, 3 Moderate: 25–50% of the fruit, and 4 Severe: >50% of the fruit. Slight epidermis defects were considered to be those allowed by the European Union Marketing Regulation for the classification of peaches and nectarines in Class I [30].

Fruit damages are of great importance in determining the potential storage period; in this work, the tolerance limits of 10% for decay and 25% for other post-harvest problems have been established [31].

#### 2.3.4. Sensory Assessment

Sensory evaluation was conducted as described by [32] with 20 judges (volunteer staff from CICYTEX-INTAEX) and regular peach consumers, who did not have any sensitivities or allergies to peaches. Judges gave informed consent before participating in the sensory trial. Samples for sensory analysis came from the other half of the peaches used for firmness and TSS measurements. Each half was divided into three portions and mixed. Each sample was prepared with three peeled portions taken at random from the sensory analysis sample set and was presented to each taster on a white plate with a three-digit code. On each day of sensory analysis, no more than three samples were tasted, with breaks between tests to avoid taster fatigue, and the mouth was rinsed with mineral water between each sample. Tasters were asked to taste each of the samples, and to mark their degree of satisfaction on a nine-point hedonic scale (1 = dislike extremely to 9 = like extremely), and the mean value was calculated [13]. Results were also expressed as the percentage of tasters whose rating was greater than 5, less than 5, and equal to 5 [33,34]. Tasters were also asked whether they would repurchase the peaches after tasting them and the percentage of affirmative or negative responses to the purchase were recorded.

### 2.4. Statistical Analysis

Statistical processing of the results was carried out with the SPSS 21.0 program (SPSS Inc, Chicago, IL, USA). For each date of storage, the results were analyzed by one-way analysis of variance (ANOVA), comparing the two harvests, and, when significant differences between means were detected, Tukey’s mean comparison test was applied (*p* < 0.05).

## 3. Results

### 3.1. Pomological Characterization of Peaches at Harvest

Table 1 shows that in ‘Rich Lady’ there were no significant differences in initial fruit characteristics between H1 and H2, which may be due to the proximity of both harvest dates, among other reasons. Both for ‘Summer Lady’ and ‘Merryl O’Henry’, H2 peaches that remained 6 and 8 days longer on the tree had significantly higher weights than those of the commercial harvest (H1).

### 3.2. Post-Harvest Evolution

#### 3.2.1. Gas Composition

The results of gas composition within the peach packages are shown in Supplementary Figure S2. As is well known, peach respiration affects different biochemical and physiological processes [19], and is affected by storage conditions, at ambient or refrigerated temperatures, and with modified or controlled atmospheres [15,18,35]. Gas concentration stabilized inside the Xtend^®^ plastic package in the first days of refrigeration. In ‘Rich Lady’, there were no significant differences between the two harvests, with the average gas concentration being 16.2% O_2_ and 2.7% CO_2_ in H1 and 15.4% O_2_ and 3.8% CO_2_ in H2; therefore, H2 peaches had a higher respiration rate, mainly at the end of the refrigerated storage. For ‘Summer Lady’, the average gas concentrations inside the packages during storage were 14.83% O_2_ and 7.21% CO_2_ for peaches of H1 and 14.64% O_2_ and 7.29% CO_2_ for H2. There were no significant differences between the two harvests, indicating similar fruit respiration rates, although the O_2_ values for H2 were a little lower inside the packages. Regarding ‘Merryl O’Henry’, H1 peaches showed a higher respiration rate, consuming a higher percentage of oxygen during refrigerated storage in PMA, with the average composition being 9.18% O_2_ and 5.32% CO_2_ for H1 and 12.51% O_2_ and 5.45% CO_2_ for H2 peaches, even though the O_2_ values for H2 were somewhat lower and more stable than those for H1 throughout refrigerated storage.

#### 3.2.2. Physical Characteristics

**Figure 1:**
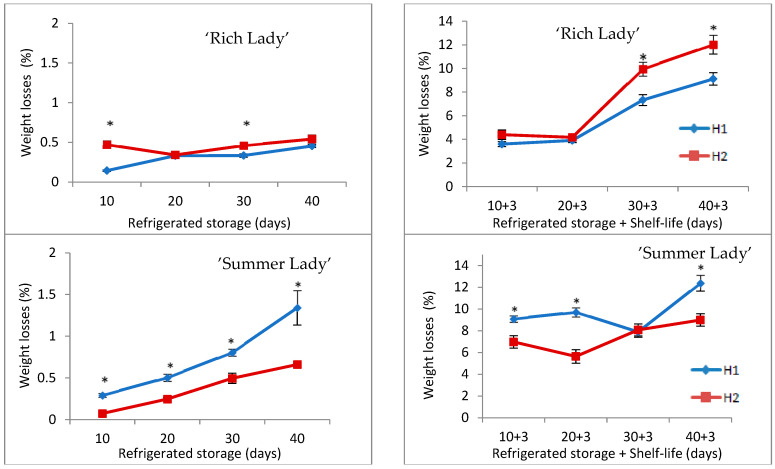

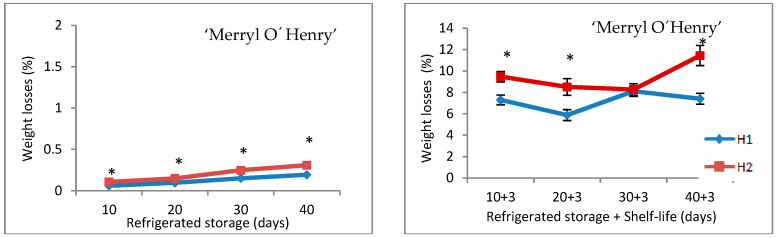
Evolution of weight losses in the three cultivars for the two harvests (H1 and H2) throughout refrigerated storage at 0 °C (**left**) and after refrigeration plus three days of shelf-life at 20 °C (**right**) (n = 30). * For each date of storage, H1 and H2 are significantly different (*p* < 0.05).

Weight losses in ‘Rich Lady’ were higher in H2 during refrigerated storage, not exceeding 0.6% in any case. Since H2 fruits had a higher respiration rate, higher transpiration was also to be expected (Figure 1). In the cultivar ‘Summer Lady’, weight loss increased as the post-harvest refrigerated storage period was prolonged. H1 fruit experienced significantly greater weight loss, always less than 1.3% at the end of refrigerated storage, which did not cause epidermis shriveling (Figure 1). Weight losses in ‘Merryl O’Henry’ peaches increased slightly during refrigerated storage, not exceeding 0.31% in any case (Figure 1), and the losses were significantly higher in H2. Out of the three cultivars studied, this was the one with the lowest weight losses during refrigerated storage. As can be seen, weight losses were not very high during refrigerated storage at 0 °C with PMA, this being one of the main known benefits of using PMA in fruit storage [14]. In peaches and nectarines, shriveling occurs when fruit weight loss exceeds 6% [36], so losses above this value set the limit for the potential storage period.

When considering total weight loss, losses during refrigeration, and shelf-life period, these were considerably higher than those during refrigerated storage. In ‘Rich Lady’ (Figure 1), there were also higher weight losses in H2, which could be related to its higher respiration rate; registering maximum values of 12%, although they did not cause visual symptoms of dehydration in the fruit. Weight losses after the shelf-life period (3 days at 20 °C) in ‘Summer Lady’ were also significantly higher in H1 (Figure 1), as it was in refrigerated storage. Total weight losses did not exceed 12.5% at any date, but fruit showed visual symptoms of dehydration as shriveling and loss of fruit volume after shelf-life, especially in H1. As in ‘Rich Lady’, weight losses were also higher in H2 for ‘Merryl O’Henry’, except at 30 + 3 days of storage, with no significant differences between H1 and H2; the highest weight loss (11.45%) was recorded in H2 at 40 + 3 days (Figure 1).

Regarding texture, firmness is a textural property that determines the evaluation of quality of fruits by consumers, and can be defined as the resistance of the material to being penetrated by a probe. Firmness is also a determining parameter limiting fruit storability. A firmness of 30 N has been set as the tolerance limit for marketing peaches in this work. With firmness between 18 and 35 N, peaches and nectarines are considered ‘ready-to-buy’ fruit and above 35 N is defined as ‘ripe and immature’ [37]. Firmness instrumentally measured is highly correlated with sensory attributes [38,39].

**Figure 2:**
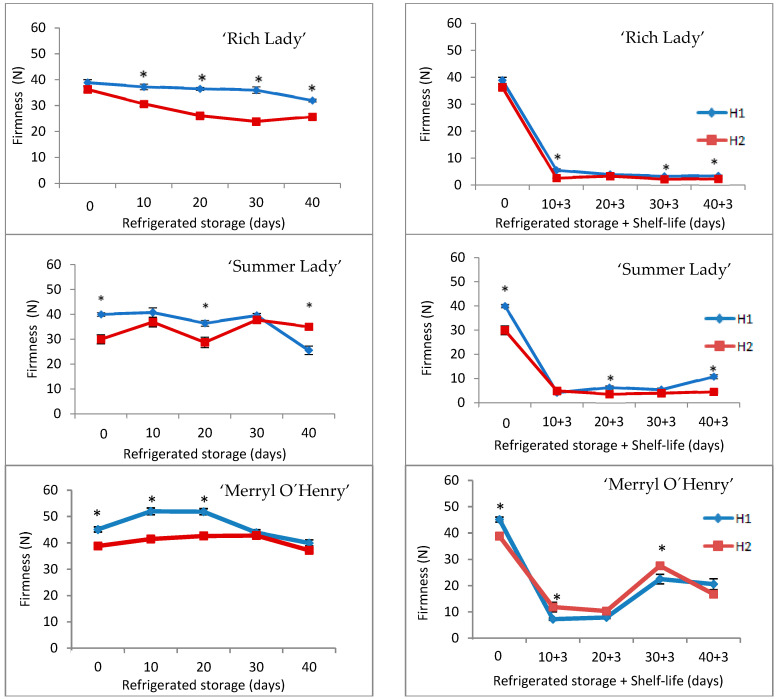
Evolution of firmness in the three cultivars for the two harvests (H1 and H2) throughout refrigerated storage at 0 °C (**left**) and after refrigeration plus three days of shelf-life at 20 °C (**right**) (n = 20). * For each date of storage, H1 and H2 are significantly different (*p* < 0.05).

As can be seen in Figure 2, ‘Rich Lady’ firmness decreased in both harvests throughout refrigerated storage, being more pronounced in H2 (29%). Peaches from H1 exceeded 30 N on all dates of analysis, while those from H2 showed firmness values below 30 N at 10 days of refrigerated storage. Considering this parameter, this cultivar should be harvested at commercial maturity (H1), since H2 peaches become too soft during refrigerated storage, limiting the fruit storability. Firmness decreased significantly after shelf-life. Considering the whole storage (refrigeration and shelf-life), it is observed that firmness values of H1 peaches were significantly higher than those of H2 for ‘Rich Lady’, except on the day of harvest and at 20 + 3 days. The lowest firmness value (2.36 N) was recorded for H2 fruits at 40 + 3 days (Figure 2).

The firmness of ‘Summer Lady’ was constant during refrigerated storage (Figure 2); at the beginning, values were significantly higher in H1 peaches, changing this trend at the end of storage. As the limit for marketing peaches has been set at 30 N, the fruits of H1 should not be stored for more than 30 days. After three days of shelf-life at 20 °C, logically, there was a considerable decrease in firmness (Figure 2), with H1 values being higher than those of H2 on all dates of analysis, with significant differences at 20 + 3 days and at 40 + 3 days of storage.

In cultivar ‘Merryl O’Henry’, firmness remained constant for up to 20 days of refrigerated storage, with H1 values being significantly higher than those of H2. From this date on, firmness decreased until the end of storage, and there were no significant differences between harvests. Peaches remained firm until the end of refrigerated storage, as only 11.26% firmness was lost in H1 and 4.22% in H2 (Figure 2). Among the three cultivars studied, ‘Merryl O’Henry’ was the one with the highest firmness values, which exceeded 30 N on all dates of analysis. As expected, after three days of shelf-life (at 20 °C), firmness decreased considerably, dropping to values close to 10 N (dates 10 + 3 and 20 + 3), increasing afterward (30 + 3 and 40 + 3), perhaps due to textural defects caused by chilling injuries (Figure 2) [11,12].

The loss of firmness is due to the degradation of insoluble protopectins to soluble pectins, so the flesh will have less firmness when the fruit ripens [40]. The enzymes that have an important role in the peach softening process are pectinmethylesterase and polygalacturonase [41].

**Figure 3:**
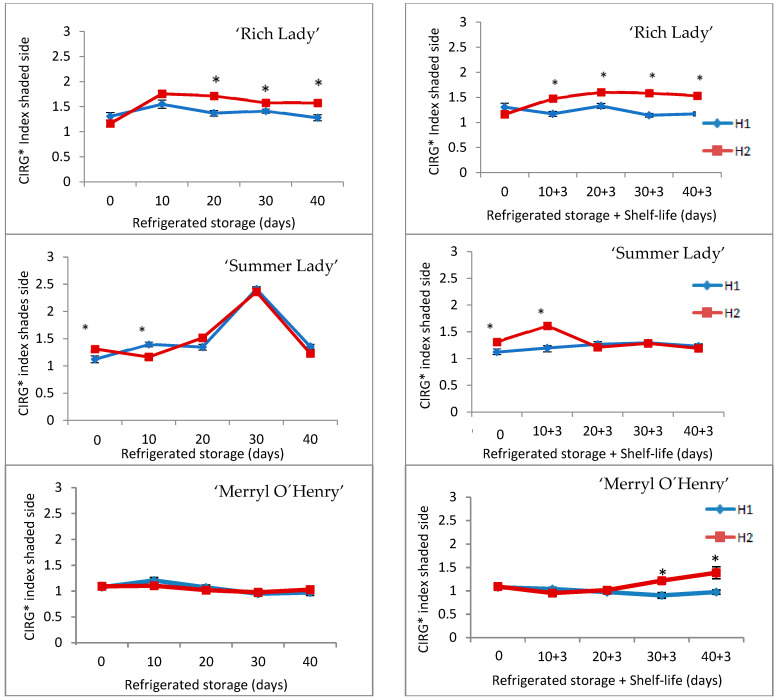
Evolution of CIRG* index of the shaded side in the three cultivars for the two harvests (H1 and H2) throughout refrigerated storage at 0 °C (**left**) and after refrigeration plus three days of shelf-life at 20 °C (**right**) (n = 20). * For each date of storage, H1 and H2 are significantly different (*p* < 0.05).

Out of the color parameters measured, only the results of the CIRG* index of the shaded side are presented, since it is the one that best reflects the evolution of the fruit during refrigeration and after shelf-life, and showed significant differences between harvests (Figure 3).

The CIRG index considers together the parameters of brightness, a* and b*, allowing us to establish with a single value the behavior of color in the fruit; it is very suitable for the study of the epidermis color in red fruit. A high CIRG index is indicative of a darker and less luminous color, while a low value corresponds to lighter and more luminous colors [28].

As can be seen in Figure 3, in ‘Rich Lady’, the CIRG parameter values remained practically stable during cold storage at 0 °C, and H2 peaches showed higher values than H1 on days 10, 20, and 30. During shelf-life, the CIRG index of the shaded side remained stable and was also significantly higher for H2 (1.6) from day 10 + 3. For the ‘Summer Lady’ cultivar, in general, the CIRG index of the epidermis remained constant throughout refrigerated storage and after shelf-life (Figure 3). Harvest date significantly affected initial color, H2 peaches having a more intense coloration. The evolution during refrigerated storage and subsequent shelf-life was very similar for fruit of both harvests, in contrast to the ‘Rich Lady’ cultivar.

The CIRG index of the shaded side remained constant in ‘Merryl O’Henry’ throughout refrigerated storage and there were no significant differences between harvests. After the shelf-life period, H1 peaches showed CIRG values similar to those of refrigerated storage, which remained constant until the end of storage. However, in H2, after 20 + 3 days of storage, the values of this index increased, showing significant differences between both harvests in the last two dates of analysis (Figure 3).

#### 3.2.3. Chemical Characteristics

**Figure 4:**
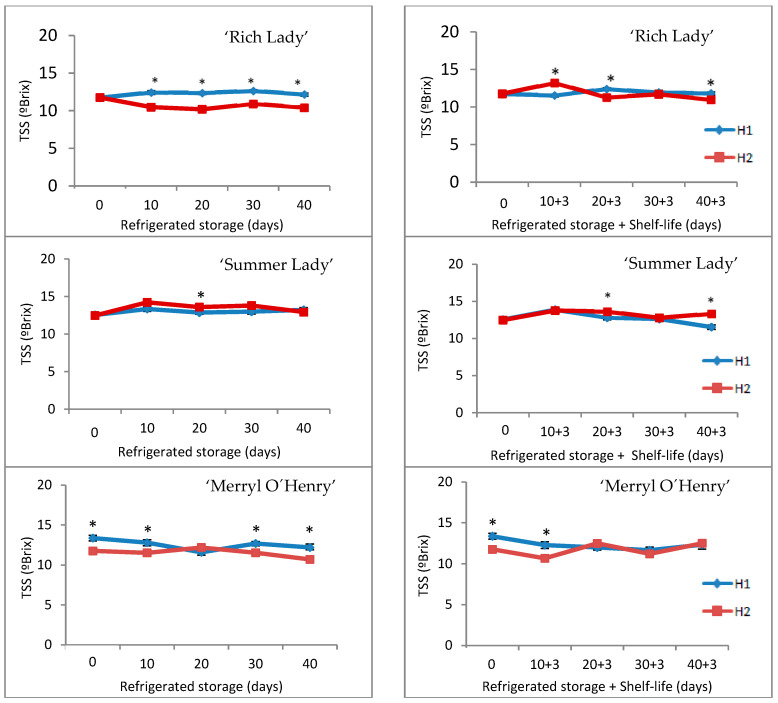
Evolution of TSSs in the three cultivars for the two harvests (H1 and H2) throughout refrigerated storage at 0 °C (**left**) and after refrigeration plus three days of shelf-life at 20 °C (**right**) (n = 20). * For each date of storage, H1 and H2 are significantly different (*p* < 0.05).

With regard to total soluble solids, ‘Summer Lady’ was the cultivar with the highest values of TSSs among the studied cultivars and their mean values of TSSs were 12.99 and 13.41 °Brix for H1 and H2, respectively (Figure 4). TSSs remained almost constant throughout refrigerated storage in all peach cultivars, and fruits of H1 showed a higher content than those of H2 in ‘Rich Lady’ (Figure 4). After the shelf-life period, TSSs are similar to those during refrigeration, and significant differences between harvests were observed especially in ‘Rich Lady’ (Figure 4). For ‘Songold’ Japanese plum, Velardo-Micharet et al. [4] found differences between harvests in the initial level of TSSs, but did not observe a variation in TSS content during post-harvest storage. In apricot, Velardo-Micharet et al. [42] observed that there was no clear effect on the evolution of TSSs during shelf-life, except for one cultivar with a slight increase. In contrast, Sang et al. [36] found that TSSs decrease during storage or shelf-life, indicating that metabolism decomposes sugar in the tissue and converts it into carbon dioxide, water, and energy. Likewise, Pan et al. [35] observed that, during the whole storage, TSSs of peach fruit all first increased, and then progressively decreased.

According to several authors [22,37,43], to increase consumer satisfaction, it is recommended that peaches have TSS values above 10 °Brix and firmness values below 35 N. In this way, taste quality is not compromised and consumer satisfaction is achieved. ‘Summer Lady’ was the cultivar with the best values for these physico-chemical characteristics.

**Figure 5:**
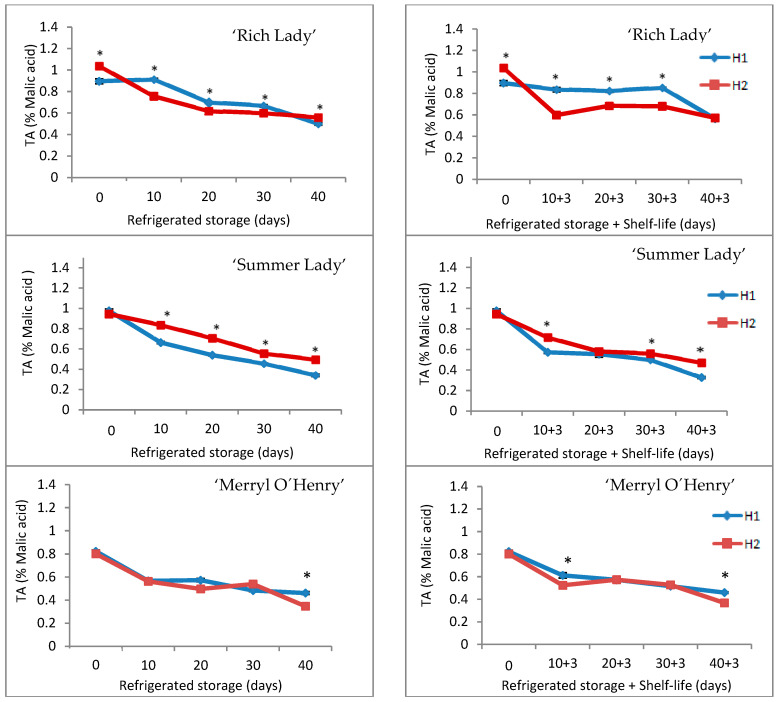
Evolution of Total Acidity in the three cultivars for the two harvests (H1 and H2) throughout refrigerated storage at 0 °C (**left**) and after refrigeration plus three days of shelf-life (**right**) (n = 5). * For each date of storage, H1 and H2 are significantly different (*p* < 0.05).

‘Rich Lady’ was the cultivar with the highest Total Acidity value at harvest, especially peaches of H2. During refrigerated storage, TA progressively decreased until the end of storage in fruits of both harvests for the three cultivars (Figure 5). This reduction was more marked for H2 fruit on the ‘Rich Lady’ cultivar and for H1 fruit on ‘Summer Lady’. The evolution of TA during the shelf-life was similar to that of refrigerated storage, with significant differences between peaches of both harvests on several dates of analysis (Figure 5).

In all cases, TA decreases during storage and shelf-life, according to other studies [14,15,17,22,33,44], because acids are used as a substrate for respiration during storage. These results are in agreement with those obtained by us for Japanese plum ‘Songold’ in prolonged storage [4], and for apricot during shelf-life [42].

**Figure 6:**
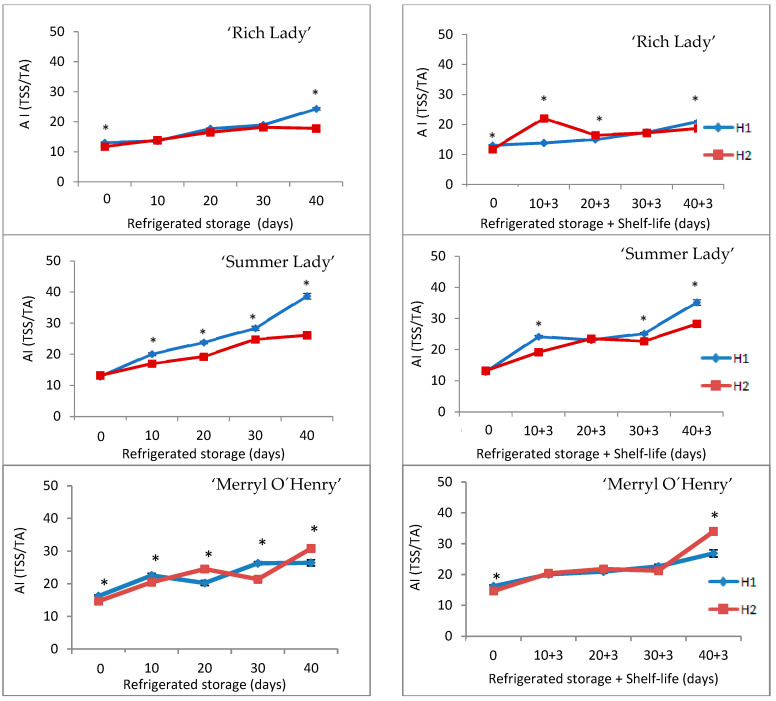
Evolution of acceptability index ratio (TSSs/TA) in the three cultivars for the two harvests (H1 and H2) throughout refrigerated storage at 0 °C (**left**) and after refrigeration plus three days of shelf-life at 20 °C (**right**) (n = 5). * For each date of storage, H1 and H2 are significantly different (*p* < 0.05).

The acceptability index ratio (AI = TSSs/TA) is of great practical importance, since it reflects the perception of organoleptic quality by consumers, according to several authors [8,43,45]. This index increased for all peach cultivars during refrigerated storage as a result of the decrease in TA although the TSS values remain almost stable. Significant differences were found between fruits of H1 and H2 in ‘Summer Lady’ and ‘Merryl O’Henry’ cultivars in all the days of analysis, and only at the beginning and at the end of refrigerated storage in ‘Rich Lady’. After the shelf-life period, the acceptability index increased progressively until the end of storage (Figure 6).

#### 3.2.4. Damage

The presence of mechanical damage (MD), including leaf rubbing, cracking, bruising, and superficial nail wounds, is an important commercial problem. Although it only affects the epidermis, it can cause losses due to dehydration and decay, as well as the acceleration of the ripening process [46], and also causes depreciation of the fruit by the purchaser. Maturity stage at harvest influences susceptibility to mechanical damage, but not all peach cultivars have the same susceptibility, and the more sensitive ones require special attention in handling. In addition, the cultivar influences susceptibility to chilling injury (CI), and it is well known that harvest date can affect CI development and susceptibility to decay, as has been pointed out for peach or nectarine [11,47] and in Japanese plum [4].

Under the conditions of the study, ‘Rich Lady’ was the most susceptible to mechanical damage after refrigerated storage and shelf-life, particularly H2 fruit (Table 2), although there was slight damage. Only H2 fruit developed decay during refrigerated storage, exceeding the tolerated limits after 20 days. No CIs were observed in this cultivar. After analyzing damage incidence in the ‘Rich Lady’ cultivar, it can be concluded that H1 peaches withstand refrigerated storage for 40 days and can be used for overseas exports. However, H2 peaches could only be used for exports that do not require more than 10 days of transport, since, at 20 + 3 days of storage, the fruits presented 10% of decay, established as the limit for their commercialization.

‘Summer Lady’ had a lower incidence of MD than ‘Rich Lady’, which was always below the established limits, and was higher in H2 peaches. H2 fruits analyzed at 20 + 3 days showed MD above the permitted limits (Table 2). Decay damage only appeared in ‘Summer Lady’ peaches after the shelf-life period, at 40 + 3 days for H1 and at 30 + 3 days for H2, higher than 10%, which is the tolerance for this type of damage. CIs appear after 40 days of refrigerated storage, mainly in H1 peaches (Table 2). These results are in agreement with those found by other authors, who emphasized the importance of the ripening stage, with the most immature fruits being the most susceptible to chilling injuries [48,49]. The CIs observed were gel and browning, with a slight and moderate level, respectively. CI was very high after shelf-life, especially on H1 peaches, with 100% of the fruit affected at 40 + 3 days. Although our study was conducted on a single season, and CI can vary from year to year, we can affirm that, in these conditions, it is advisable to harvest ‘Summer Lady’ peaches at maturity H2 (6 days after H1), and transport time should not exceed 20 days, due to the high incidence of CI.

In the fruits of ‘Merryl O’Henry’, the percentage of MD observed during refrigeration was low and its intensity level was slight, being slightly higher in H1; after the shelf-life period, a higher percentage of MD was observed in H2, although below the tolerance limit (Table 2). Decay did not appear in this cultivar until the shelf-life period. It is noteworthy that, in H1, only on one date of analysis (30 + 3 days) was 5% rot observed. In H2, this damage was greater than in H1 and higher than the 10% limit from 30 + 3 days, which limits the commercialization of these peaches. CIs were only detected at the last date of analysis for both H1 and H2 during refrigerated storage, slightly higher for H1. After shelf-life, CI percentage was very high, particularly in H2 peaches. CI incidence limits the commercial life of this cultivar. It should be harvested at commercial maturity (H1) and used for exports requiring a maximum of 10 days of transport. In contrast to what was observed for ‘Sumer Lady’ and what was found by others [48,49], in ‘Merryl O’Henry’, the more immature fruits were less susceptible to CI. Although CI is a characteristic of the cultivar, studies by Campos-Vargas et al. [10] suggest that seasonal variations should be considered to define the severity and incidence of damage, since the same trials on ‘Merryl O’Henry’ peaches carried out in two different geographical areas showed different results. It would be interesting to conduct future studies to determine the factors that mainly affect CI development in this cultivar, or to apply pre-ripening protocols, which has been shown to be effective in reducing the incidence of chilling injury [22].

#### 3.2.5. Sensory Evaluation

Table 3 shows mean values of the judges’ scores for the three peach cultivars, during refrigerated storage and after shelf-life. Results of the taster acceptance rating are shown in Supplementary Figure S3. For statistical analysis, samples were compared throughout storage, separately for each cultivar and each harvest after refrigerated storage and after a 3-day shelf-life.

‘Merryl O’Henry’ was the worst rated of the three cultivars, both for H1 and H2 fruits. After shelf-life, peaches showed a high incidence of damage, and H2 peaches at 30 + 3 and 40 + 3 days and H1 peaches at 40 + 3 days were not in a proper condition to be tasted by the judges. The ‘Summer Lady’ cultivar was, in general, the best rated. ‘Rich Lady’ scored lowest at harvest day (0) in both H1 and H2. However, after refrigeration and shelf-life, peaches of this cultivar achieved good scores, especially at 10 + 3 and 20 + 3 days. It is this moment, when peaches would be consumed at home, after a trip of up to 20 days, which allows for proper ripening.

Our results are in agreement with those of Giné-Bordonaba et al. [13], who found that, in general, consumer acceptability was mainly influenced by cultivar and storage, and increased during shelf-life at 20 °C. On the other hand, cold storage resulted in lower consumer acceptance for most cultivars but, again, except for ‘Big Top’ [13].

**Figure 7:**
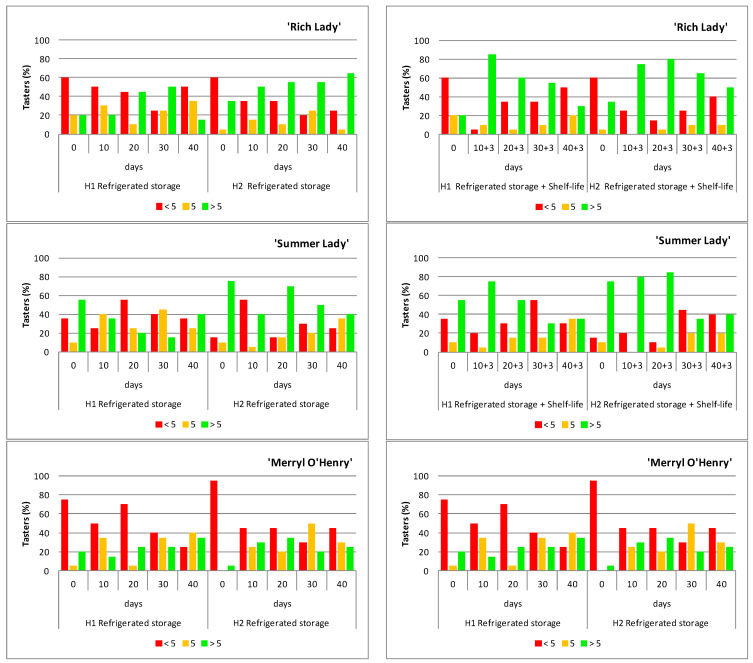
Percentage of tasters assigning scores below 5 (red bar), equal to 5 (orange bar), and above 5 (green bar) in the sensory evaluation of the three peach cultivars, of harvests H1 and H2, throughout refrigerated storage at 0 °C (**left**) and after refrigeration plus three days of shelf-life at 20 °C (**right**). (n = 20).

Figure 7 shows the frequency with which tasters rated samples with a score less than 5 (red bar), equal to 5 (orange bar), and greater than 5 (green bar) for the three peach cultivars ‘Rich Lady’, ‘Summer Lady’, and ‘Merryl O’Henry’, of harvests H1 and H2, during refrigerated storage and after shelf-life.

The tasters’ ratings of the ‘Rich Lady’ cultivar after the refrigerated storage period (Figure 7) show that this cultivar was poorly rated on the day of harvest, for H1 and H2, with a high percentage of scores below 5, which may be due to the unripe state of the peaches at the time of harvest. However, as the days of refrigerated storage elapsed, the scores assigned by the tasters were higher, in the case of H1 until day 30 and of H2 until the end of storage. The ‘Rich Lady’ cultivar was best rated at H2, as the percentage of scores above 5 was higher at harvest and on all storage dates. This indicates that, although the TSS percentage of the fruit remained constant throughout storage, the decrease in acidity and firmness pleased the tasters. It is particularly worthy of note that, after 40 days of refrigerated storage, the H2 fruit still scored very well, while the H1 fruit scored very poorly.

After the refrigerated storage and shelf-life period (Figure 7), the percentage of tasters who rated this cultivar with scores above 5 remained higher in H2. Moreover, the positive scores (above 5) in H1 started to decrease earlier (10 + 3 days) than in H2 (20 + 3 days). In H2 at 10 + 3 days of storage, where there was a pronounced drop in acidity, 75% of the tasters rated these peaches with scores of 7, 8, and 9 points, which means that this drop in acidity was perceived by the tasters.

It is important to note that ‘Summer Lady’, unlike ‘Rich Lady’, showed good acceptance from the day of harvest (Figure 7) and was the best-rated of the three cultivars under study.

The ratings were higher on H2 fruit, since on all storage dates the percentage of ratings higher than 5 was higher than on H1, except on the last storage date, where it was equal on both. In H1, the percentage of grades higher than 5 decreases from the harvest day, and in H2, this decrease did not occur until 20 days of storage. Concerning the tasters’ ratings after the refrigeration plus shelf-life period, the results are shown in Figure 7. There was an increase in the rating of the fruits of both H1 and H2, after the shelf-life period, with respect to the fruits just removed from storage. On the other hand, sensory rating after shelf-life presented a similar trend to that of refrigeration, i.e., scores in H1 began to decrease at 10 + 3 days; in H2, this did not occur until 20 + 3 days.

The ratings of ‘Merryl O’Henry’ (Figure 7) show that it was the worst evaluated of the three cultivars studied, mainly due to its high degree of immaturity and high incidence of chilling injury. After the shelf-life period, the ratings improved slightly with respect to those obtained during refrigerated storage; however, in none of the cases, the percentage of scores above 5 exceeded 45% (Figure 7). These peaches were unable to be tasted at every storage date due to excessive browning of the fruit.

In the sensory analysis of the ‘Merryl O’Henry’ cultivar on the different dates of refrigerated storage of both harvests, H1 and H2, there was a very high percentage of scores below 5. This indicates that it was a poorly rated cultivar, and the notes made by the tasters indicated that the fruit was immature, tasteless, hard, and woody.

In general, H2 peaches were rated higher by tasters, although this is not related to physical–chemical parameters, as the acceptability index is better for H1. Thus, sensory analysis is essential in post-harvest studies to assess fruit quality. Although some parameters, such as TSSs, TA, or AI, show adequate values, other aspects, such as CI or decay, may be taken into account by tasters, so that sensory assessment may not correspond to the evolution of standard quality.

**Figure 8:**
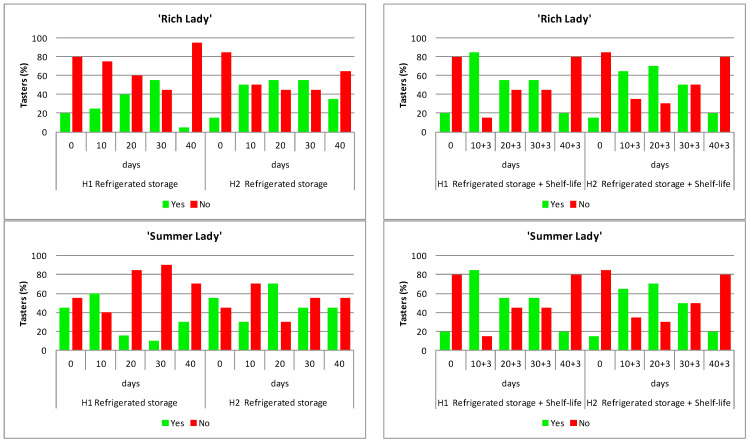

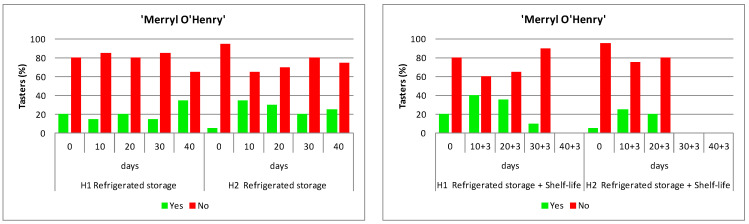
Percentage of tasters’ choice to purchase for the three peach cultivars ‘Rich Lady’, ‘Summer Lady’, and ‘Merryl O’Henry’, of harvests H1 and H2, throughout refrigerated storage at 0 °C (**left**) and after refrigeration plus three days of shelf-life at 20 °C (**right**). (n = 20). Green color bars mean yes and red bars mean no.

As mentioned above, the tasters were also asked if they would purchase the peaches again, and the percentage of affirmative or negative responses to the decision was recorded, as shown in Figure 8.

For ‘Rich Lady’, the percentage of tasters that responded “Yes” to the question “Would buy this cultivar again?”, was in accordance with the evaluations obtained in the tasting, being higher in H2 (Figure 8). The purchase option corresponds with the sensory evaluation, i.e., as the scores of H1 increased, the percentage of tasters who would buy this cultivar increased until day 30 of storage under refrigeration. Only 5% of tasters would buy these peaches after 40 days of storage, which means that this cultivar should not be marketed after being stored for more than 30 days under refrigeration; even if the fruit maintains an adequate firmness and a good appearance, the consumer would not buy it. However, H2 fruit after 40 days of refrigerated storage would be bought again by 35% of tasters.

The tasters’ assessment of the fruit after refrigerated storage and shelf-life is shown in Figure 8.

The overall percentage of tasters who would buy this cultivar was higher in H1. For peaches from H2, it can be observed that the percentage of tasters who would buy this cultivar increased until 20 + 3 days of storage, after which it started to decrease.

The results of the tasters’ option to buy ‘Summer Lady’ during refrigerated storage and the subsequent shelf-life are shown in Figure 8 as a total percentage of tasters who would or would not (Yes/No) buy this peach cultivar. On harvest date, the percentage of tasters who would purchase was higher for H2 fruit. The percentage of tasters who would repeat purchases decreased after 10 days of storage for H1, and after 20 days for H2; it is important to point out that, on all storage dates, the percentage of repeat purchases was higher for peaches from H2. After the shelf-life period (3 days at 20 °C), the total percentage of tasters who would purchase this cultivar was higher than that obtained under refrigeration and was also higher for H2.

Concerning ‘Merryl O’Henry’ for all storage dates, both during refrigeration (Figure 8) and after shelf-life (Figure 8), the percentage of tasters who would not buy this cultivar was higher than those who would buy it, and the difference was very marked, thus once again reflecting the rejection of tasters for this cultivar.

## 4. Conclusions

‘Rich Lady’ cultivar fruits of H1 can withstand refrigerated storage for 40 days, and therefore, under the conditions of our study, would be suitable for export overseas. Although sensory evaluation of H2 fruit was better, the lack of firmness and the incidence of decay, exceeding the tolerated limits, would not allow them to be marketed to distant markets.

For ‘Summer Lady’, H1 peaches are not suitable for markets requiring more than 10 days of transport, under these experimental conditions. It is advisable to delay the harvest date by 6 days (H2), as this will improve sensory quality and post-harvest storage capacity. The limiting factor is the high incidence of chilling injury, which is revealed (>10%) during shelf-life after 20 days of refrigerated storage.

The cultivar ‘Merryl O’Henry’ has high firmness and susceptibility to chilling injury for both H1 and H2, with low tasters’ acceptability scores and choice to purchase in all cases. Delaying the harvest date does not improve sensory quality and increases susceptibility to chilling injury. The limitation of this study is that it was carried out in only one season. For this cultivar, it would be advisable to study the application of pre-ripening treatments to improve post-harvest quality and reduce the incidence of chilling injury.

## Figures and Tables

**Table 1:** Characterization of the starting fruit of ‘Rich Lady’, ‘Summer Lady’, and ‘Merryl O’Henry’ peaches at commercial harvest (H1) and after 5, 6, and 8 days (H2), respectively.

	‘Rich Lady’		‘Summer Lady’		‘Merryl O’Henry’	
Harvest	H1	H2	H1	H2	H1	H2
Size (mm)	61.46 ± 2.22	61.75 ± 2.06	70.98 ± 3.22	72.16 ± 2.61	72.26 ± 2.92 *	75.58 ± 2.18 *
Weight (g)	119.03 ± 10.88	120.66 ± 9.51	176.17 ± 18.37 *	188.24 ± 16.28 *	195.24 ± 19.41 *	223.66 ± 15.15 *
Length (mm)	58.81 ± 3.25	57.60 ± 2.67	63.71 ± 3.51	65.10 ± 3.19	67.96 ± 3.03	70.31 ± 3.90

Mean values ± Standard Deviation (*n* = 30). By parameter measured, * within the same cultivar indicates significant differences (*p* < 0.05).

**Table 2:** Damage incidence (%) in ‘Rich Lady’, ‘Summer Lady’, and ‘Merryl O’Henry’ for both harvests, during refrigerated storage, and after shelf-life.

Harvest	Date	‘Rich Lady’			‘Summer Lady’			‘Merryl O’Henry’		
		MD	D	CI	MD	D	CI	MD	D	CI
Refrigerated storage										
H1	10	20	0	0	10	0	0	5	0	0
	20	35	0	0	5	0	0	5	0	0
	30	25	0	0	10	0	0	5	5	0
	40	10	0	0	5	0	30	20	0	15
H2	10	10	0	0	15	0	0	5	0	0
	20	30	15	0	20	0	0	5	0	0
	30	15	0	0	20	0	0	5	0	0
	40	5	10	0	5	0	5	5	0	5
Refrigerated storage + Shelf-life										
H1	10 + 3	60	0	0	0	0	10	15	0	0
	20 + 3	20	0	0	10	0	60	15	0	30
	30 + 3	20	0	0	20	0	70	0	5	55
	40 + 3	15	30	0	20	15	100	10	0	85
H2	10 + 3	20	10	0	15	0	0	10	0	45
	20 + 3	55	0	0	50	5	0	0	5	75
	30 + 3	45	25	0	15	15	80	30	35	80
	40 + 3	20	95	0	0	0	10	25	30	75

MD: Mechanical damage; D: Decay; CI: Chilling injury.

**Table 3:** Mean values ± Standard Deviation of tasters’ acceptability scores * for ‘Rich Lady’, ‘Summer Lady’, and ‘Merryl O’Henry’ of both harvests, during refrigerated storage and after shelf-life.

Harvest	Date	‘Rich Lady’	‘Summer Lady’	‘Merryl O’Henry’
Refrigerated storage				
H1	0	4.00 ± 1.52 b	5.35 ± 1.50 a	4.15 ± 1.27 a
	10	4.70 ±1.42 ab	5.05 ± 1.39 a	4.20 ± 1.61 a
	20	4.95 ± 1.32 ab	4.25 ± 1.29 a	4.10 ± 1.80 a
	30	5.30 ± 1.08 a	4.60± 1.10 a	4.80 ± 1.11 a
	40	4.40 ± 1.23 ab	4.85 ± 1.69 a	5.05 ± 0.89 a
H2	0	4.35 ± 1.53 b	6.05 ± 1.50 a	3.30 ± 0.86 b
	10	5.05 ± 1.39 ab	4.40 ± 1.79 b	4.65 ± 1.42 a
	20	5.50 ± 1.64 ab	6.20 ± 1.61 a	4.80 ± 1.24 a
	30	5.70 ± 1.26 a	5.40 ± 1.43 ab	5.05 ± 1.23 a
	40	4.75 ± 1.33 ab	5.20 ± 1.36 ab	4.70 ± 1.30 a
Refrigerated storage + Shelf-life				
H1	10 + 3	6.50 ± 1.05 a	5.75 ± 1.33 a	5.30 ± 1.56 a
	20 + 3	5.55 ± 1.85 ab	5.45 ± 1.85 a	4.65 ± 1.46 a
	30 + 3	5.30 ± 1.87 ab	4.40± 1.76 a	4.75 ± 0.79 a
	40 + 3	4.30 ± 1.53 b	4.65 ± 1.69 a	--
H2	10 + 3	6.00 ± 1.78 a	6.45 ± 1.67 a	5.10 ± 1.83 a
	20 + 3	6.20 ± 1.44 a	6.55 ± 1.28 a	4.40 ± 1.50 a
	30 + 3	5.80 ± 1.58 a	4.55 ± 1.39 b	--
	40 + 3	5.25 ± 1.52 a	5.00 ± 1.65 b	--

Mean values ± Standard Deviation (n = 20). * Nine-point hedonic scale (1 = dislike extremely to 9 = like extremely). Values followed by different letters differ at a significance level of 0.05; according to Tukey’s test, samples were compared throughout storage, separately for each cultivar and each harvest (H1 and H2), after refrigerated storage at 0 °C and after refrigeration plus three days of shelf-life at 20 °C.

## Data Availability

The original contributions presented in this study are included in the article and Supplementary Materials. Further inquiries can be directed to the corresponding author.

## Supplementary Materials

The following supporting information can be downloaded at https://www.mdpi.com/article/10.3390/foods15030421/s1, Figure S1: Photographs of the three peach cultivars at harvest; Figure S2: Evolution of gas composition inside PMA packages for cultivars ‘Rich Lady’ (A), ‘Summer Lady’ (B), and ‘Merryl O’Henry’ (C) of the two harvests, H1 and H2 (n = 3). Figure S3: Number of tasters assigning scores below 5 (1 to 4, red bars), equal to 5 (orange bar), and above 5 (6–9, green bars) in the sensory evaluation of the three peach cultivars ‘Rich Lady’, ‘Summer Lady’, and ‘Merryl O’Henry’, of harvests H1 and H2, during refrigerated storage and after shelf-life.
