# Supplementary material for: Effect of Harvest Date on Fruit Quality and Post-Harvest Storability of Three Different Peach Cultivars
Source: Foods. 2026 Jan 23;15(3):421. doi: 10.3390/foods15030421 (PMC12896964; doi:10.3390/foods15030421)
Supplement: Supplementary file 1 [file foods-15-00421-s001.zip › Figure S2.pdf]

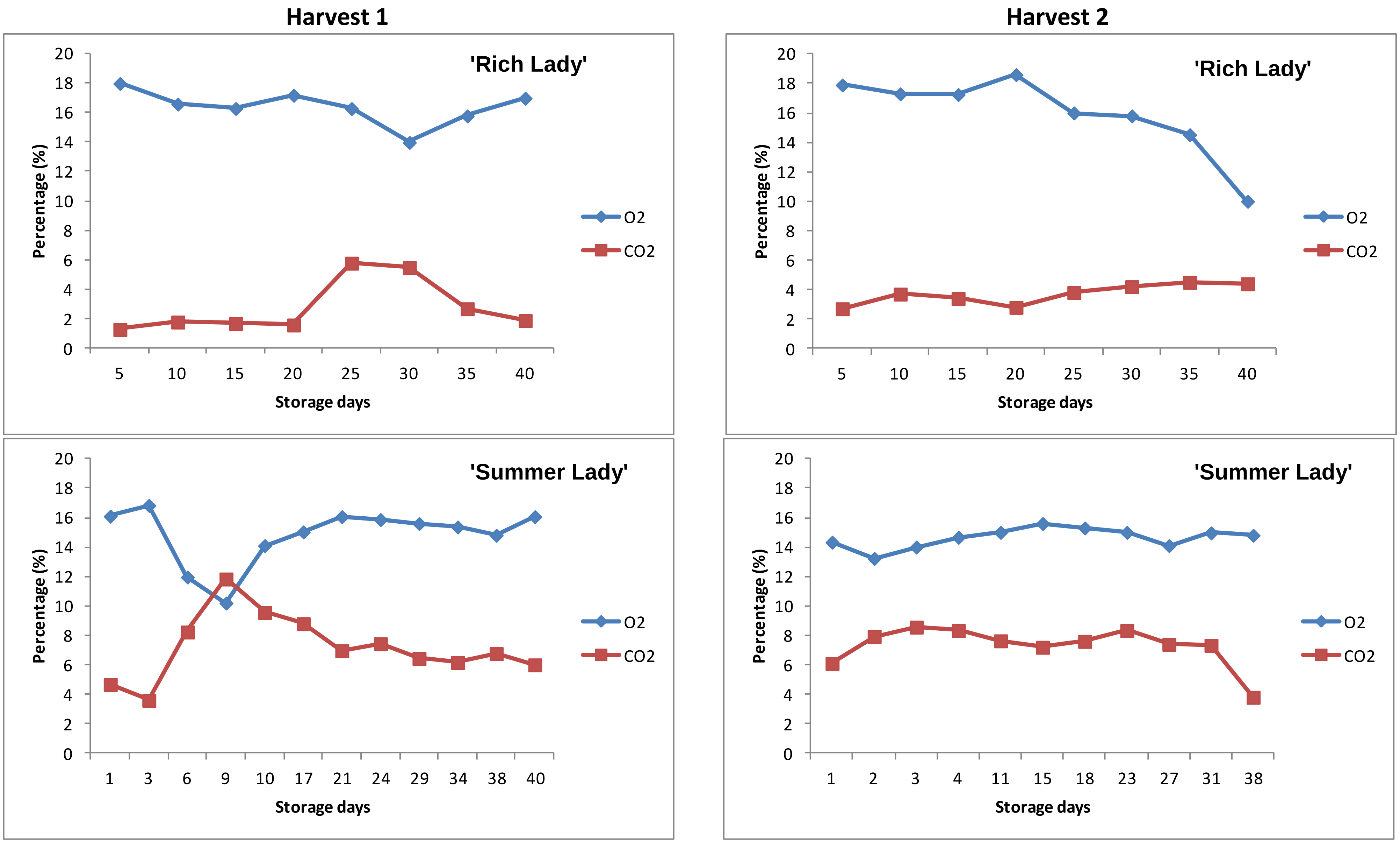
Harvest 1
Harvest 2
'Rich Lady'
'Rich Lady'
'Summer Lady'
'Summer Lady'
Percentage (%)
Storage days
O2
CO2
0
2
4
6
8
10
12
14
16
18
20
5
10
15
20
25
30
35
40
1
3
6
9
10
17
21
24
29
34
38
40
1
2
3
4
11
15
18
23
27
31
38

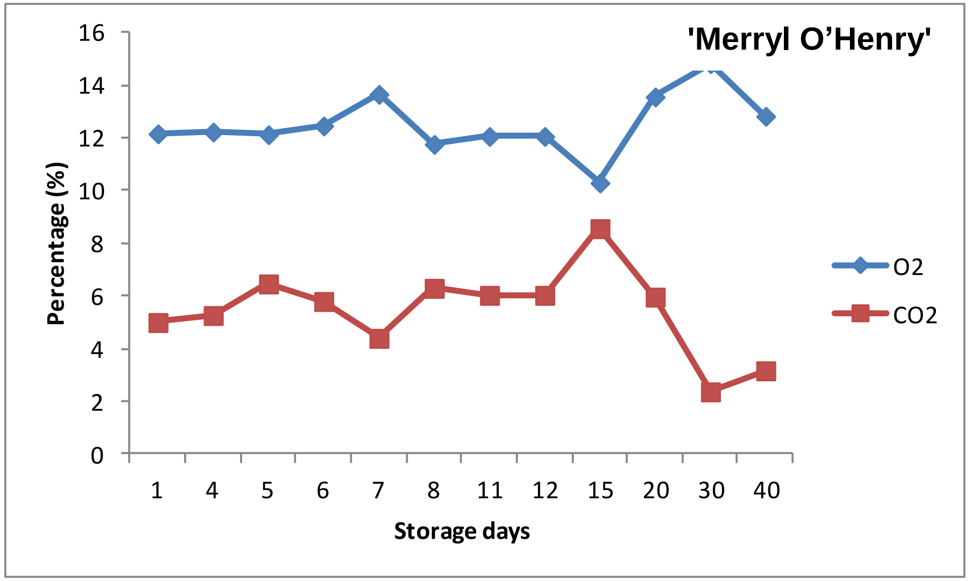

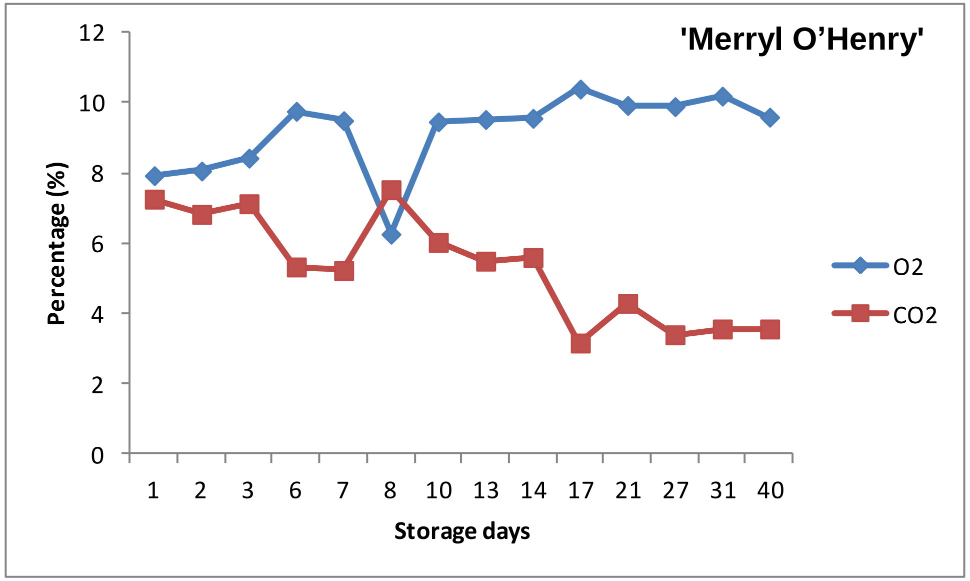

Figure S2. Evolution of gas composition inside PMA packages for cultivars 'Rich Lady' (A), 'Summer Lady' (B), and 'Merryl O´Henry' (C) of the two harvests, H1 and H2 (n=3).
