# Supplementary material for: Effect of Harvest Date on Fruit Quality and Post-Harvest Storability of Three Different Peach Cultivars
Source: Foods. 2026 Jan 23;15(3):421. doi: 10.3390/foods15030421 (PMC12896964; doi:10.3390/foods15030421)
Supplement: Supplementary file 1 [file foods-15-00421-s001.zip › Figure S3.pdf]

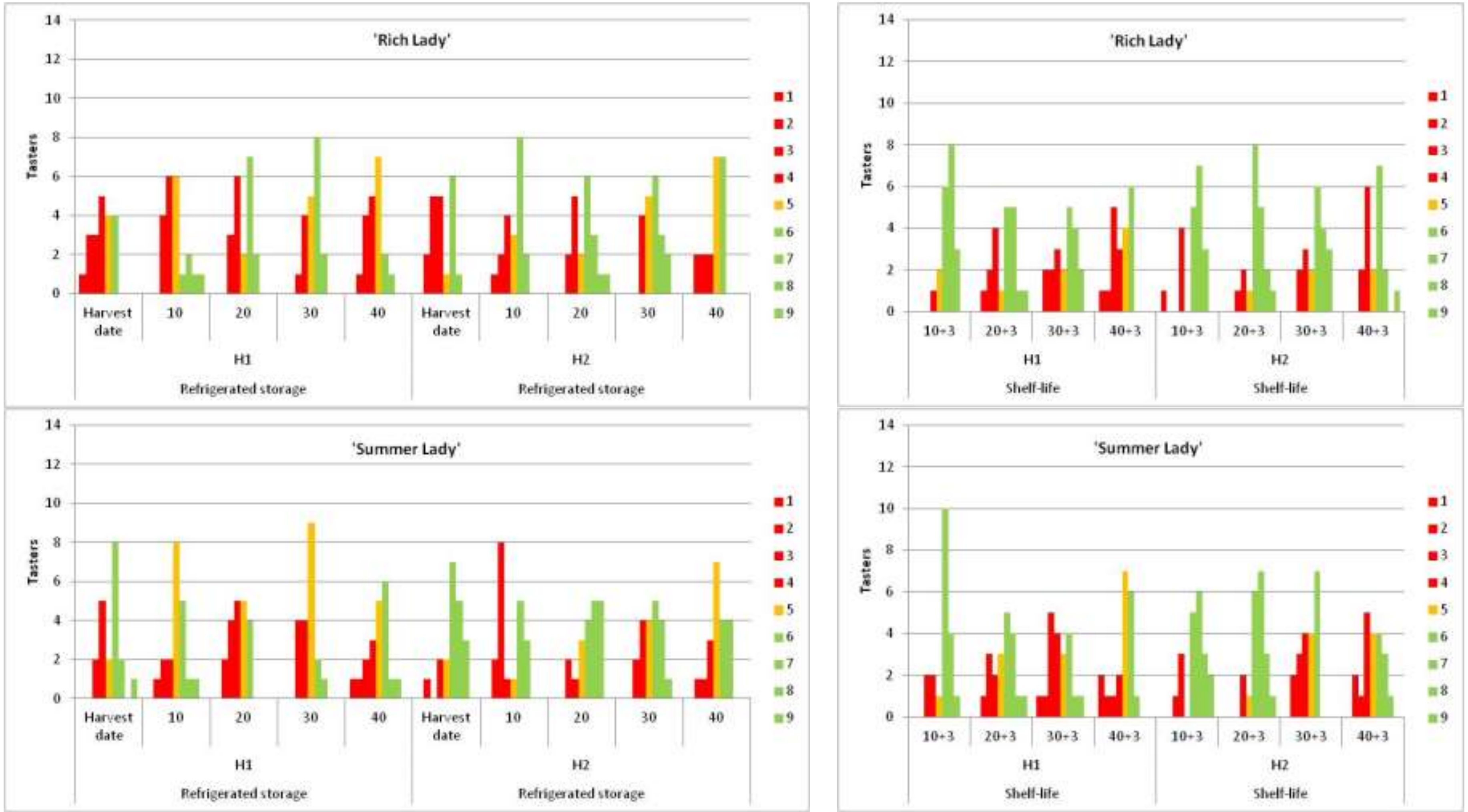
'Rich Lady'
Tasters
14
12
10
8
6
4
2
0
Harvest date
10
20
30
40
H1
H2
Refrigerated storage
1
2
3
4
5
6
7
8
9
'Rich Lady'
10+3
20+3
30+3
40+3
Shelf-life
'Summer Lady'
'Summer Lady'

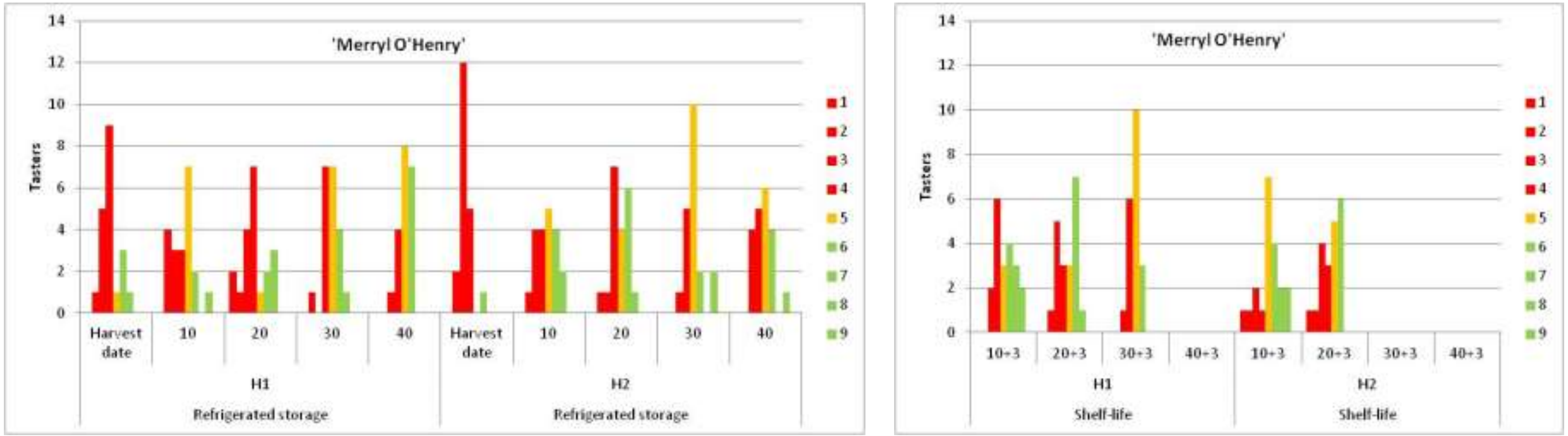

Figure S3. Number of tasters assigning scores below 5 (1 to 4, red bars), equal to 5 (orange bar) and above 5 (6-9 green bars) in the sensory evaluation of the three peach cultivars 'Rich Lady', ‘Summer Lady’, and ‘Merryl O´Henry’, of harvests H1 and H2, during refrigerated storage and after shelf-life.
