# Supplementary figures and images for: Effect of Harvest Date on Fruit Quality and Post-Harvest Storability of Three Different Peach Cultivars
Source: Foods. 2026 Jan 23;15(3):421. doi: 10.3390/foods15030421 (PMC12896964; doi:10.3390/foods15030421)

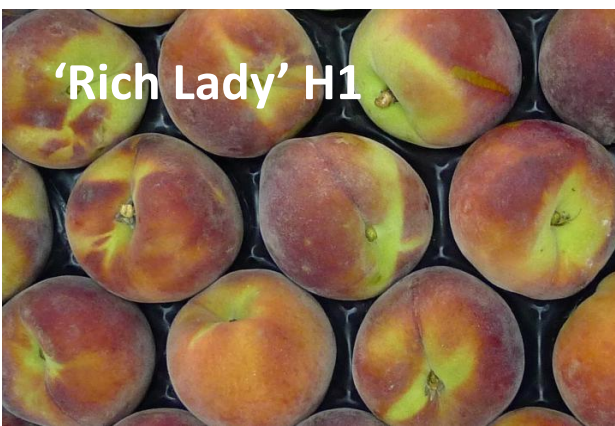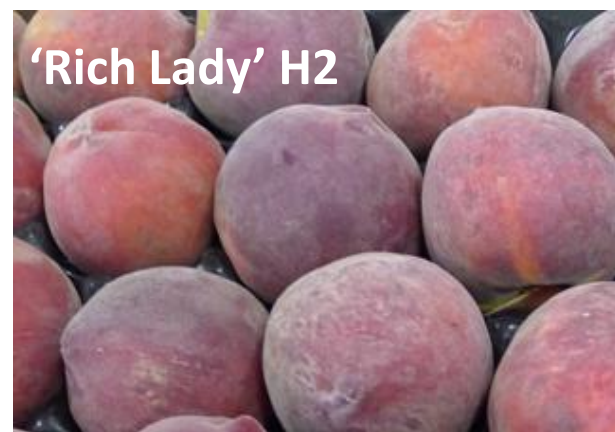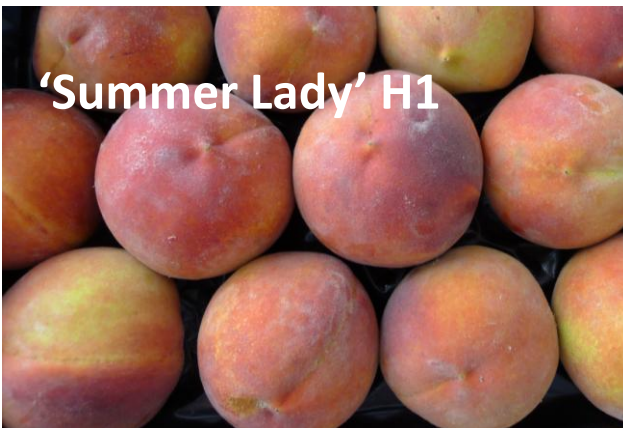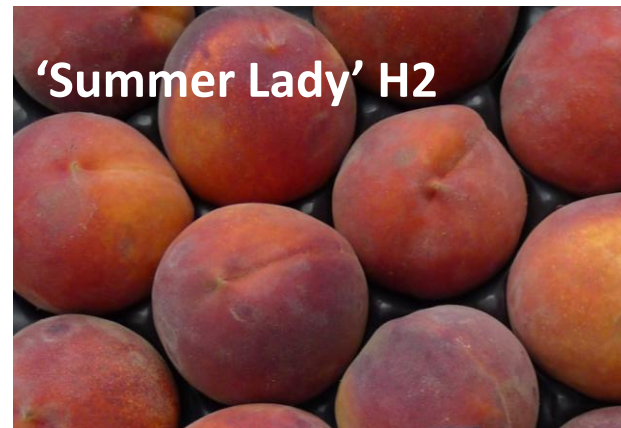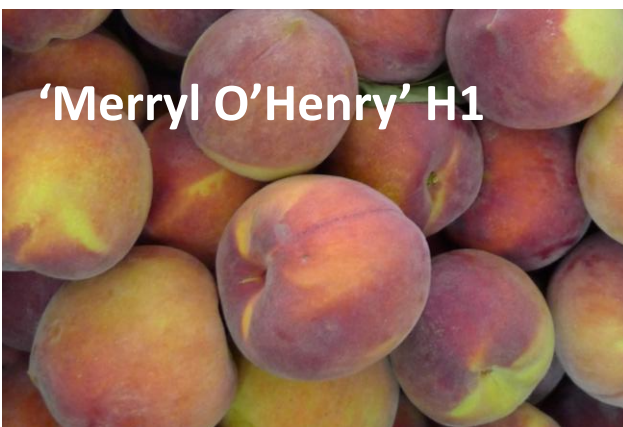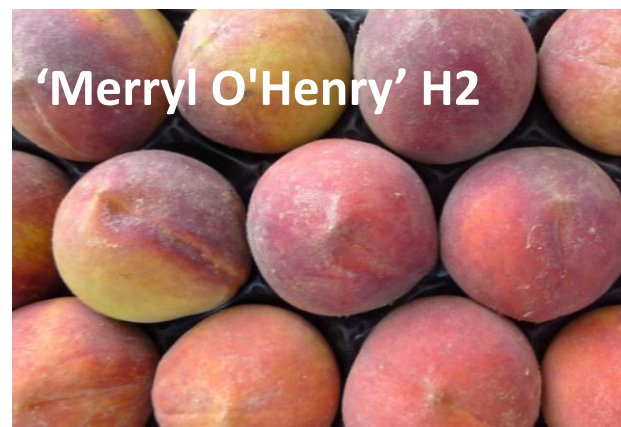

**Figure S1.** Photographs of the three peach cultivars at harvests H1 and H2

Supplement: Supplementary file 1 [file foods-15-00421-s001.zip › Figure S1.pdf]
